# Lyssavirus Nucleoprotein Induces GPAT4‐Mediated CYB5R1 Depalmitoylation to Suppress Ferroptosis and Promote Viral Replication

**DOI:** 10.1002/advs.77672

**Published:** 2026-09-09

**Authors:** Jianqing Zhao, Chuan Liang, Pingping Chen, Meixin Sun, Zhen F. Fu, Ling Zhao, Ming Zhou

**Affiliations:** ^1^ National Key Laboratory of Agricultural Microbiology Huazhong Agricultural University Wuhan China; ^2^ Key Laboratory of Preventive Veterinary Medicine of Hubei Province College of Veterinary Medicine Huazhong Agricultural University Wuhan China; ^3^ Frontiers Science Center for Animal Breeding and Sustainable Production Wuhan China; ^4^ Hubei Hongshan Laboratory Wuhan China

**Keywords:** CYB5R1, ferroptosis, GPAT4, Lyssavirus, palmitoylation

## Abstract

Rabies virus (RABV) and other *lyssaviruses* exploit lipid droplet (LD) formation to evade host defenses, but the underlying mechanisms remain unclear. Here, we demonstrate that *lyssavirus* N proteins induce LD biogenesis by upregulating glycerol‐3‐phosphate acyltransferase 4 (GPAT4) expression and promoting its translocation to the LD surface, a conserved mechanism across the *lyssavirus* genus. Mechanistically, GPAT4‐mediated LD formation sequesters free fatty acids, leading to acyl‐protein thioesterase 1 (APT1)‐dependent depalmitoylation of NADH‐cytochrome B5 reductase 1 (CYB5R1) at Cys208 and Cys278. This post‐translational modification triggers autophagic degradation of CYB5R1, thereby impairing its ability to induce ferroptosis via two complementary pathways: nuclear receptor co‐activator 4 (NCOA4)‐mediated ferritinophagy and H_2_O_2_ production. Conversely, diacylglycerol O‐acyltransferase (DGAT) inhibitors or GPAT4 knockdown restores CYB5R1 palmitoylation and stability, reinstates ferroptosis, and suppresses RABV infection. Our findings reveal a novel “*lyssavirus* N‐GPAT4‐LD‐CYB5R1 palmitoylation” axis that modulates ferroptosis susceptibility, highlighting protein palmitoylation as a critical regulatory node in virus‐host interactions and identifying GPAT4 as a potential antiviral target.

## Graphical abstract

Lipid metabolism plays a vital role in viral infections. Here, we reveal that the lyssavirus N protein regulates GPAT4 to induce lipid droplet biogenesis, restricting CYB5R1 palmitoylation and reducing ferroptosis susceptibility to ultimately promote viral replication. This N‐GPAT4‐LD‐CYB5R1 axis underscores protein palmitoylation as a key regulatory node in virus‐host interactions.

## 1 Introduction

*Lyssaviruses* are neurotropic rhabdoviruses that cause fatal encephalitis in mammals, with 17 recognized species grouped into two phylogroups [1, 2]. Phylogroup I includes Rabies virus (RABV), the most extensively studied *lyssavirus*, alongside Australian bat *lyssavirus* (ABLV) and Duvenhage virus (DUVV), while phylogroup II comprises Lagos bat virus (LBV) [3]. The *lyssavirus* genome encodes five structural proteins: nucleoprotein (N), phosphoprotein (P), matrix protein (M), glycoprotein (G), and large polymerase (L), with the N protein emerging as a key regulator of viral replication and host adaptation [4].

Lipid droplets (LDs), dynamic organelles storing neutral lipids (triacylglycerols, TGs; cholesteryl esters), are critical for lipid homeostasis in the central nervous system [5]. Neurons, with limited antioxidant capacity, rely on LDs to sequester excess free fatty acids, reducing oxidative stress and lipotoxicity [6, 7]. Viral infections, including those by neurotropic viruses, induce LD biogenesis to support replication and suppress host defenses. The ORF6 protein of coronaviruses interacts with the endoplasmic reticulum (ER) membrane proteins BAP31 and USE1 to mediate the formation of ER‐LD contacts. Concurrently, it interacts with the SAM complex in the outer mitochondrial membrane, tethering mitochondria to LDs to facilitate ATP transport for viral replication. Additionally, our previous study has indicated that neuroinvasive viruses induce LD formation to restrict Bcl‐2‐mediated apoptosis, thereby facilitating their replication. Although the relationship between viral infection and LD formation has been deeply investigated [8, 9, 10], however, the molecular mechanisms linking virus‐induced LD formation to neuronal survival and ferroptosis remain poorly defined.

Ferroptosis, an iron‐dependent, lipid peroxidation‐driven cell death pathway, has emerged as an antiviral defense mechanism in certain viral infections, including porcine epidemic diarrhea virus (PEDV), infectious spleen and kidney necrosis virus (ISKNV) and hepatitis C virus [11, 12, 13, 14]. Intracellular ferrous iron (Fe^2+^) is either utilized in mitochondrial metabolism or stored in ferritin, with excess Fe^2+^ fueling Fenton reactions to generate lipid‐damaging reactive oxygen species (ROS) [15, 16, 17]. Under iron deficiency, nuclear receptor co‐activator 4 (NCOA4) mediates ferritinophagy‐lysosomal degradation of ferritin to release Fe^2+^ and maintain iron homeostasis [18, 19, 20, 21]. While ferroptosis is recognized as a host antiviral strategy, how neurotropic viruses like *lyssaviruses* evade this pathway remains unclear.

Post‐translational modifications (PTMs) are central to virus‐host interactions, S‐palmitoylation, the reversible attachment of palmitic acid to cysteine residues, emerging as a key regulatory node [22, 23]. Dynamic palmitoylation modulates protein localization, stability, and function, and its dysregulation by viruses is increasingly linked to immune evasion [24, 25]. Cytochrome b5 reductase 1 (CYB5R1), an electron‐transfer enzyme, has been implicated in ferroptosis via H_2_O_2_ production [26], yet how PTMs regulate CYB5R1 during viral infection, and whether this impacts *lyssavirus* replication, remains unknown.

Here, we identify a conserved mechanism by which *lyssaviruses* hijack lipid metabolism to suppress ferroptosis through CYB5R1 depalmitoylation. We demonstrate that *lyssavirus* N proteins induce LD biogenesis by upregulating glycerol‐3‐phosphate acyltransferase 4 (GPAT4), sequestering palmitic acid to drive APT1‐mediated CYB5R1 depalmitoylation at Cys208/Cys278. This triggers autophagic degradation of CYB5R1, impairing its ability to induce ferroptosis via NCOA4‐ferritinophagy and H_2_O_2_ production. Our findings reveal a novel “virus‐lipid‐PTM” regulatory axis and highlight GPAT4 as a potential antiviral target.

## 2 Results

### 2.1 Restriction of Lipid Droplet Formation via DGAT Inhibitors Suppresses RABV Infection Through CYB5R1 Upregulation

**FIGURE 1:**
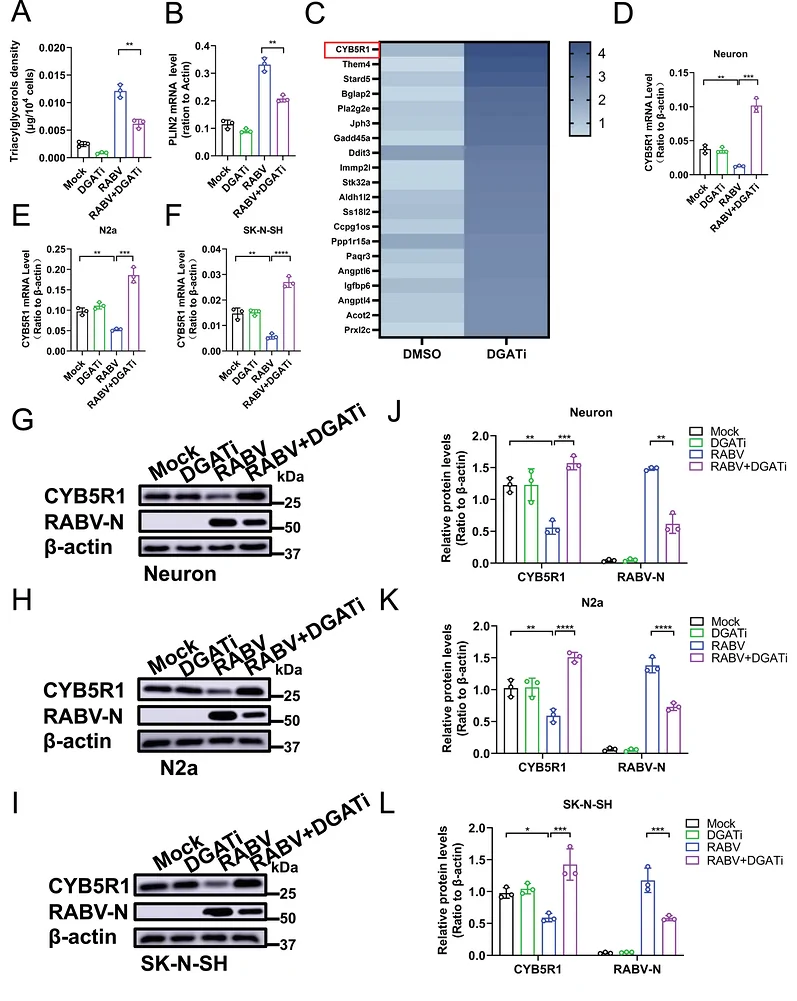
Inhibition of Triglyceride Biosynthesis Restores CYB5R1 Expression in Lyssavirus‐Infected Neuronal Cells. (A) Cellular TG levels and (B) PLIN2 mRNA expression in N2a cells infected with RABV (MOI = 1, 48 h) and treated with DGAT inhibitors (A922500+PF06424439; 8 µg/mL + 10 µg/mL) or DMSO. (C) RNA‐seq heatmap showing differentially expressed genes (DEGs) in RABV‐infected N2a cells ± DGAT inhibitors. CYB5R1 mRNA levels in (D) primary neurons, (E) N2a cells, and (F) SK‐N‐SH cells infected with RABV ± DGAT inhibitors. Immunoblot analysis of CYB5R1 and RABV‐N protein levels in (G) primary neurons, (H) N2a cells and (I) SK‐N‐SH cells under identical treatments. (J‐L) Quantification of immunoblots in (G‐I). Representative results from three independent experiments are shown. Data are expressed as mean±SD, n = 3. Statistical significance was determined using unpaired two‐tailed student's *t*‐test. *p < 0.05, **p < 0.01, ***p < 0.001, ****p < 0.0001.

Our previous work demonstrated that RABV elevates cellular TG levels by enhancing diacylglycerol O‐acyltransferase (DGAT) activity, thereby inducing LD formation, and this process promotes viral replication by suppressing neuronal ferroptosis. However, the molecular mechanisms by which LDs regulate ferroptosis at the transcriptional or post‐translational level during RABV infection remain unclear. To address this knowledge gap, we first confirmed that RABV infection increases both TG levels and the expression of PLIN2 (a LD surface protein) in N2a cells. Notably, treatment with DGAT inhibitors (targeting DGAT1/2) significantly reduced these RABV‐induced elevations (Figure 1A,B). We then performed RNA‐seq to profile transcriptomic changes in RABV‐infected cells following DGAT inhibition, which identified NADH‐cytochrome B5 reductase 1 (CYB5R1) as the most significantly upregulated gene (Figure 1C). To validate this finding, we examined CYB5R1 mRNA expression in three neuronal models: primary neurons, N2a cells, and the human neuroblastoma cell line SK‐N‐SH. In all models, RABV infection significantly decreased CYB5R1 mRNA levels, whereas DGAT inhibition reversed this effect (Figure 1D–F). Consistently, CYB5R1 protein levels were reduced by RABV infection but rescued by DGAT inhibitor treatment (Figure 1G–L). Concomitantly, DGAT inhibitor treatment decreased RABV‐N protein levels in infected neuronal cells, confirming that LD inhibition restricts RABV infection. Taken together, these results establish that restricting LD formation via DGAT inhibitors suppresses RABV infection through the upregulation of CYB5R1.

### 2.2 CYB5R1 Suppresses RABV Infection by Inhibiting Viral Replication in Neuronal Cells

**FIGURE 2:**
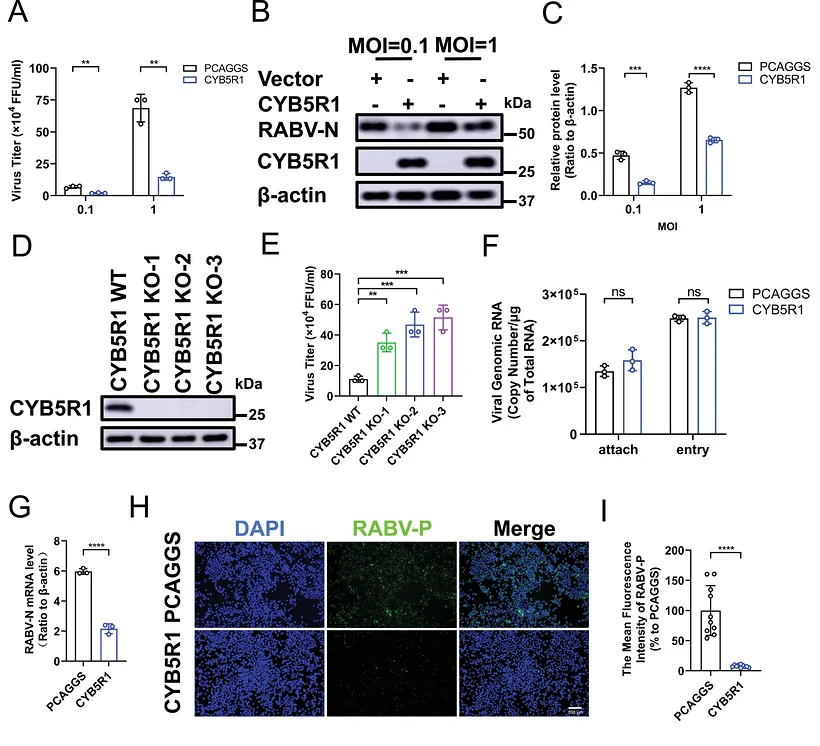
CYB5R1 restricts RABV production through inhibiting viral replication. (A) Viral titers in N2a cells transfected with CYB5R1‐HA or empty vector (PCAGGS) and infected with RABV (MOI = 0.1/1, 48 h). (B‐C) Immunoblot analysis (B) and quantification (C) of RABV‐N protein levels in CYB5R1‐overexpressing N2a cells. (D) Immunoblot validation of CYB5R1‐KO in N2a cells. (E) Viral titers in CYB5R1‐KO N2a cells infected with RABV (MOI = 1, 48 h). (F) RABV genomic RNA levels (attachment/entry phase: 4°C for 1 h or 37°C for 2 h, MOI = 1). (G) RABV‐N mRNA levels (replication phase: 24 h post‐infection, MOI = 1). (H‐I) Immunofluorescence analysis of RABV‐P protein (green) in CYB5R1‐overexpressing N2a cells (H, scale bar: 100 µm, n = 10 cells for each group) and quantification of fluorescence intensity (I). Representative results from three independent experiments are shown. Data are expressed as mean±SD, n = 3. Statistical significance was determined using unpaired two‐tailed student's t‐test or one‐way ANOVA followed by Tukey's multiple comparisons test. **p < 0.01, ***p < 0.001, ****p < 0.0001, ns: not significant.

To investigate whether CYB5R1 modulates RABV infection, we first overexpressed CYB5R1 in N2a cells, followed by RABV infection at varying MOIs, and then quantified viral titers. Overexpression of CYB5R1 significantly reduced both RABV viral titer (Figure 2A) and RABV‐N protein expression (Figure 2B,C). Conversely, CRISPR/Cas9‐mediated knockout of CYB5R1 (CYB5R1‐KO) in N2a cells (Figure 2D) resulted in a significant increase in RABV viral titer compared to wild‐type cells (Figure 2E). To further define the stage at which CYB5R1 acts, we analyzed its impact on key steps of the RABV life cycle: attachment, entry, and replication. Viral genomic RNA levels did not differ significantly between the CYB5R1‐overexpressing and empty vector control groups (Figure 2F), indicating CYB5R1 does not affect RABV attachment or entry. In contrast, during the replication stage, CYB5R1 overexpression led to a significant reduction in RABV‐N mRNA levels (Figure 2G). Consistently, immunofluorescence staining with anti‐RABV‐P antibody revealed decreased RABV fluorescence intensity in CYB5R1‐overexpressing N2a cells (Figure 2H,I). Taken together, these findings demonstrate that CYB5R1 suppresses RABV infection primarily by inhibiting viral replication.

### 2.3 CYB5R1 Restricts RABV Replication by Modulating Ferroptosis Susceptibility Through NCOA4‐Mediated Ferritinophagy and H_2_O_2_ Production

CYB5R1 has been reported to induce ferroptosis by catalyzing H_2_O_2_ production [26]. Based on this, we hypothesized that CYB5R1 may restrict RABV replication by modulating ferroptosis susceptibility. To test this, we first examined whether CYB5R1 promotes ferroptosis during RABV infection. Overexpression of CYB5R1 in N2a cells followed by RABV infection significantly increased cell death (Figure S1A), as measured by propidium iodide staining, and led to elevated levels of hallmark ferroptosis markers: cellular malondialdehyde (MDA), ferrous ion (Fe^2+^) accumulation, and lipid peroxidation (Figure S1B–D). These effects were recapitulated in RABV‐infected SK‐N‐SH cells overexpressing CYB5R1 (Figure S1E–H), confirming that CYB5R1 enhances ferroptosis during RABV infection. Notably, CYB5R1 overexpression specifically increased intracellular Fe^2+^ levels in RABV‐infected N2a cells, suggesting that CYB5R1 modulates ferroptosis susceptibility primarily through regulation of cellular iron metabolism. Meanwhile, treatment with Ferrostatin‐1, the specific ferroptosis inhibitor, inhibited the enhancement of ferroptosis mediated by CYB5R1 during RABV infection in both N2a and SK‐N‐SH cells (Figure S1I,J). Furthermore, inhibiting LD biogenesis in CYB5R1‐KO N2a cells resulted in a significantly attenuated suppression of viral replication compared to wild‐type (WT) N2a cells (Figure S2A). Concurrently, both lipid peroxidation and Fe^2+^ levels exhibited a marked increase (Figure S2B,C).

Given our prior observation that DGAT inhibitors upregulate CYB5R1 in RABV‐infected N2a cells, we further investigated whether DGAT inhibition alters iron metabolism under these conditions. We analyzed the expression of key iron metabolism regulatory genes, including Transferrin receptor, Divalent metal transporter 1, Iron‐Responsive Element Binding Proteins 1/2, ferroportin, Ferritin Heavy/Light Chain, and NCOA4. Among these, Ferritin Heavy Chain (FTH) expression was significantly reduced, whereas NCOA4 expression was significantly increased (Figure S3A). Consistently, RABV infection alone increased NCOA4 protein levels and decreased FTH protein levels in N2a cells, and these trends were further exacerbated by DGAT inhibitor treatment (Figure S3B,C).

**FIGURE 3:**
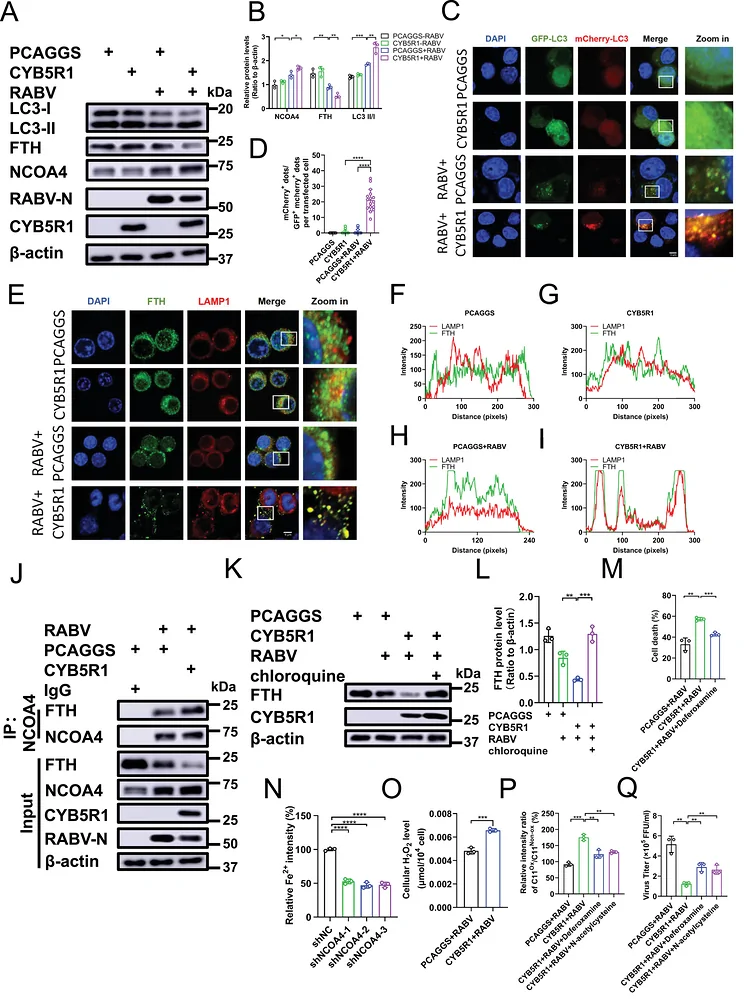
CYB5R1 modulates neuronal Ferroptosis via NCOA4‐Mediated Ferritinophagy and H_2_O_2_ Production. Immunoblot analysis (A) and quantification (B) of LC3, FTH and NCOA4 in CYB5R1‐transfected N2a cells infected with RABV (MOI = 1, 48 h). Representative images (C) and quantification (D) of mcehrry‐LC3 puncta/mCherry‐GFP‐LC3 puncta in N2a cells (scale bar: 5 µm, n = 16 cells for each group). Representative images (E) and co‐localization analysis (F‐I) of FTH (green) and LAMP1 (red) (E, scale bar: 5 µm). (J) Co‐IP analysis of endogenous NCOA4‐FTH interaction in CYB5R1‐overexpressing N2a cells. (K‐L) Immunoblot analysis (K) and quantification (L) of FTH in N2a cells treated with chloroquine (10 µM, autophagy inhibitor). (M) Cell death in CYB5R1‐overexpressing cells treated with deferoxamine (100 µM). (N) Intracellular Fe^2+^ levels in N2a cells transfected with shNCOA4. (O) H_2_O_2_ production in CYB5R1‐overexpressing N2a cells. (P) Lipid peroxidation in cells treated with Deferoxamine or N‐acetylcysteine (0.5 mM). (Q) Viral titers in N2a cells under indicated treatments. Representative results from three independent experiments are shown. Data are expressed as mean±SD, n = 3. Statistical significance was determined using unpaired two‐tailed student's t‐test or one‐way ANOVA followed by Tukey's multiple comparisons test. *p < 0.05, **p < 0.01, ***p < 0.001, ****p < 0.0001, ns: not significant.

NCOA4 mediates FTH degradation via ferritinophagy [19, 27]. Based on this, we hypothesized that CYB5R1 restricts RABV replication by inducing ferroptosis through ferritinophagy. As predicted, CYB5R1 overexpression in RABV‐infected N2a cells increased the LC3II/I ratio and NCOA4 expression, both established autophagy markers, decreased FTH expression, and concomitantly reduced RABV‐N protein levels (Figure 3A,B). Consistent with this, the ratio of mCherry^+^ puncta (autolysosomes) was significantly elevated in CYB5R1‐overexpressing RABV‐infected N2a cells (Figure 3C,D), confirming that CYB5R1 induces ferritinophagy in RABV‐infected neuronal cells. To define the degradation pathway, we observed enhanced colocalization of FTH with LAMP1 (lysosomal marker) and strengthened NCOA4‐FTH interaction in CYB5R1‐overexpressing RABV‐infected N2a cells (Figure 3E–J), suggesting lysosomal‐dependent FTH degradation. This was validated using chloroquine, a ferritinophagy inhibitor [20], which rescued CYB5R1‐induced FTH degradation (Figure 3K,L). We next investigated whether CYB5R1 modulates ferroptosis via iron metabolism. Deferoxamine‐mediated iron chelation restored cell viability in CYB5R1‐overexpressing RABV‐infected N2a cells (Figure 3M), while NCOA4 knockdown via shRNA reduced intracellular Fe^2+^ levels (Figure 3N), indicating CYB5R1 promotes ferroptosis by increasing labile iron.

Consistent with prior reports that CYB5R1 catalyzes H_2_O_2_ production to induce ferroptosis [26], CYB5R1 overexpression increased H_2_O_2_ levels in RABV‐infected N2a cells (Figure 3O). Inhibition of Fe^2+^ (Deferoxamine) or H_2_O_2_ (N‐acetylcysteine) reduced lipid peroxidation (Figure 3P) and rescued RABV replication (Figure 3Q). Together, these findings demonstrate that CYB5R1 restricts RABV replication by enhancing ferroptosis susceptibility through two complementary mechanisms: NCOA4‐mediated ferritinophagy and H_2_O_2_ production.

### 2.4 RABV‐N Reduces CYB5R1 Stability by Promoting APT1‐Mediated Depalmitoylation via Inducing LD Formation

**FIGURE 4:**
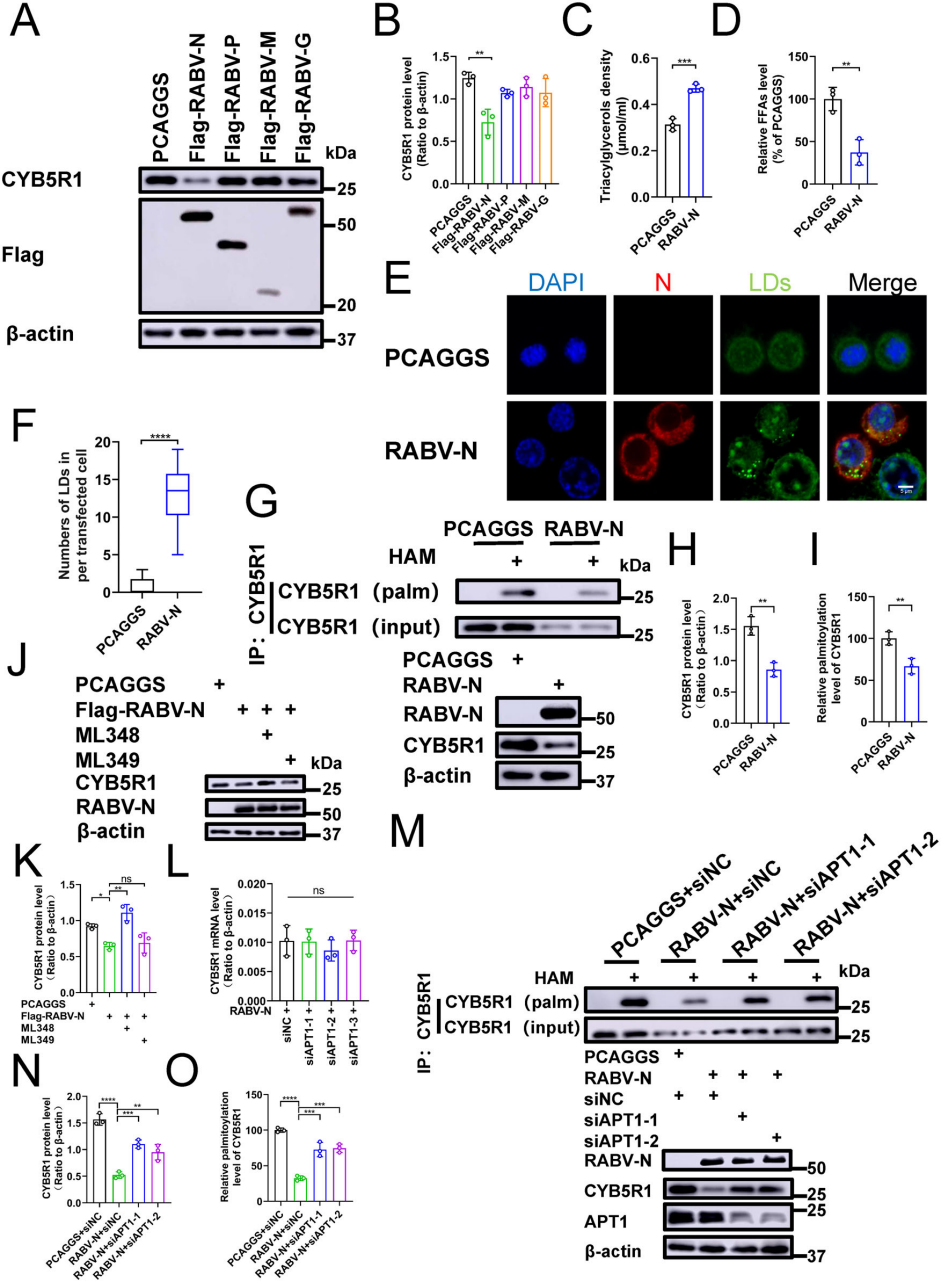
RABV‐N increases LD formation to induce CYB5R1 depalmitoylation via APT1. (A) Immunoblot analysis and (B) quantification of CYB5R1 protein levels in N2a cells transfected with RABV structural proteins (N/P/M/G). (C) Cellular TG and (D) free fatty acid levels in RABV‐N‐transfected N2a cells. LD staining in RABV‐N‐transfected N2a cells (E, scale bar: 5 µm) and quantification of LD number (F, *n* = 24 cells for each group). (G) Immunoblot analysis, quantification of CYB5R1 (H) expression and (I) palmitoylation in RABV‐N transfected N2a cells. (J) Immunoblot analysis and (K) quantification of endogenous CYB5R1 protein levels in N2a cells treated with APT1 inhibitor (ML348, 10 µM) or APT2 inhibitor (ML349, 10 µM). (L) Quantification of CYB5R1 mRNA expression in RABV‐N transfected N2a cells with siAPT1 transfecting. (M) Immunoblot analysis, quantification of CYB5R1 (N) expression and (O) palmitoylation in RABV‐N transfected N2a cells with cotransfecting siNC or siAPT1. Representative results from three independent experiments are shown. Data are expressed as mean±SD, *n* = 3. Statistical significance was determined using unpaired two‐tailed student's *t*‐test. *p < 0.05, **p < 0.01, ***p < 0.001, ****p < 0.0001, ns, not significant.

To investigate whether CYB5R1 downregulation is mediated by RABV structural proteins, we individually transfected N2a cells with plasmids expressing RABV N, P, M, or G protein. Only RABV‐N expression significantly reduced endogenous CYB5R1 protein levels (Figure 4A,B). Given our prior observation that DGAT inhibitor‐induced LD depletion upregulates CYB5R1, we hypothesized that RABV‐N may modulate CYB5R1 expression by altering LD synthesis. Consistent with this, RABV‐N transfection in N2a cells increased TG levels and LD number, while decreasing free fatty acid levels (Figure 4C–F), confirming that RABV‐N promotes LD formation in neuronal cells. Our previous study demonstrated that RABV‐induced LDs sequester palmitic acid for triglyceride synthesis [28]. Since palmitic acid must be converted into palmitoyl‐CoA to serve as an acyl donor in protein S‐palmitoylation [29, 30]. We therefore hypothesized that RABV‐N‐induced LDs may reduce CYB5R1 protein levels by impairing its palmitoylation. First, to test whether RABV infection restricts CYB5R1 palmitoylation by reducing cytosolic palmitoyl‐CoA, we measured its levels and found a significant decrease upon RABV infection; conversely, inhibiting LD biogenesis with DGATi further increased palmitoyl‐CoA levels (Figure S4).

Furthermore, RABV‐N transfection in N2a cells decreased both CYB5R1 protein expression and palmitoylation levels (Figure 4G–I). Moreover, to identify the depalmitoylase involved, we treated cells with ML348 (APT1 inhibitor) or ML349 (APT2 inhibitor). ML348, but not ML349, restored endogenous and exogenous CYB5R1 protein stability (Figures 4J,K and S5A,B). ML348 treatment increased CYB5R1 protein levels in a dose‐dependent manner without affecting its mRNA transcription (Figure S5C–E). Similarly, APT1 knockdown did not alter CYB5R1 mRNA levels but significantly increased CYB5R1 protein expression and palmitoylation (Figure 4L–O).

Previous studies have shown that depalmitoylated APT1 is predominantly localized in the cytosol, where it catalyzes the depalmitoylation of other target proteins [31, 32]. Therefore, we hypothesized that RABV‐N might induce LD biogenesis to inhibit APT1 palmitoylation, thereby promoting its cytosolic accumulation to exert depalmitoylase activity on CYB5R1 To investigate this, we examined cytosolic APT1 levels in RABV‐N‐transfected cells, with or without DGATi treatment to restrict LD formation. Results indicated that RABV‐N transfection significantly increased cytosolic APT1 levels in N2a cells, whereas DGATi‐mediated LD inhibition effectively reversed this effect (Figure S5F,G). Furthermore, treatment with the palmitoylation inhibitor 2‐BP resulted in a dose‐dependent increase in cytosolic APT1 levels (Figure S5H,I), further corroborating our hypothesis. Together, these results indicate that RABV‐N reduces CYB5R1 stability by promoting APT1‐mediated depalmitoylation.

### 2.5 RABV‐N Induces Autophagic Degradation of CYB5R1 by Reducing Palmitoylation at Cys208 and Cys278 to Decrease Ferroptosis Susceptibility

**FIGURE 5:**
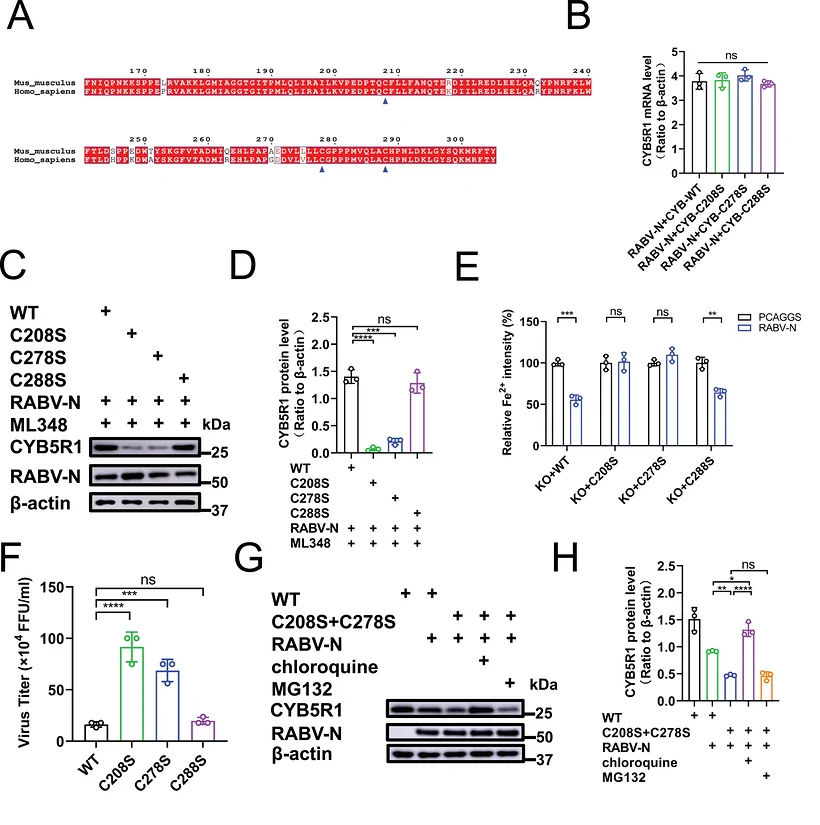
RABV‐N reduces CYB5R1 stability via APT1‐mediated depalmitoylation at Cys208 and Cys278. (A) Schematic of CYB5R1 with putative palmitoylation sites (Cys208/Cys278/Cys288). (B) CYB5R1 mRNA levels in N2a cells transfected with CYB5R1 WT or C208S/C278S/C288S mutants. (C) Immunoblot analysis and (D) quantification of CYB5R1 mutants in N2a cells treated with APT1 inhibitor (ML348, 10 µM). (E) Intracellular Fe^2+^ levels in CYB5R1‐KO N2a cells transfected with RABV‐N and CYB5R1 mutants. (F) Viral titers in CYB5R1‐KO N2a cells transfected with CYB5R1 mutants and infected with RABV (MOI = 1, 48 h). (G) Immunoblot analysis and (H) quantification of CYB5R1 mutants in N2a cells treated with MG132 (10 µM, proteasome inhibitor) or chloroquine (10 µM). Representative results from three independent experiments are shown. Data are expressed as mean±SD, *n* = 3. Statistical significance was determined using unpaired two‐tailed student's t‐test or one‐way ANOVA followed by Tukey's multiple comparisons test. **p < 0.01, ***p < 0.001, ****p < 0.0001, ns: not significant.

We further identified the key palmitoylation sites of CYB5R1 by screening its amino acid sequence, which revealed three cysteine residues at positions 208, 278, and 288 (Figure 5A). These residues were individually mutated to serine (C208S, C278S, C288S). While CYB5R1 mRNA levels in N2a cells transfected with these three mutants remained unchanged (Figure 5B), ML348‐mediated APT1 inhibition failed to stabilize CYB5R1 protein in the C208S and C278S mutants (Figure 5C,D). These results indicate that Cys208 and Cys278 are critical for CYB5R1 palmitoylation, and RABV‐N induces CYB5R1 depalmitoylation at these sites to reduce its stability. To assess whether CYB5R1 palmitoylation modulates ferroptosis susceptibility, we transfected CYB5R1‐KO N2a cells with wild‐type CYB5R1 or its C208S, C278S, or C288S mutants, followed by RABV‐N transfection or RABV infection. Unlike wild‐type CYB5R1, the C208S and C278S mutants failed to restore RABV‐N‐induced Fe^2+^ reduction (Figure 5E) and viral restriction (Figure 5F), indicating that depalmitoylation at Cys208 and Cys278 blocks CYB5R1‐mediated Fe^2+^ accumulation and ferroptosis susceptibility. Next, we investigated the degradation pathway of depalmitoylated CYB5R1. Chloroquine (an autophagy inhibitor), but not MG132 (a proteasome inhibitor), rescued CYB5R1 degradation in C208S+C278S mutants (Figure 5G,H), confirming that depalmitoylated CYB5R1 is degraded via autophagy. Consequently, RABV replication was rescued in cells expressing these mutants (Figure 5H). Together, these findings demonstrate that RABV‐N reduces CYB5R1 palmitoylation at Cys208 and Cys278, triggering autophagic degradation of CYB5R1 and thereby decreasing cellular susceptibility to ferroptosis.

### 2.6 RABV‐N Induces GPAT4 Expression and Translocation to LD Surface to Promote LD Formation and Maturation

**FIGURE 6:**
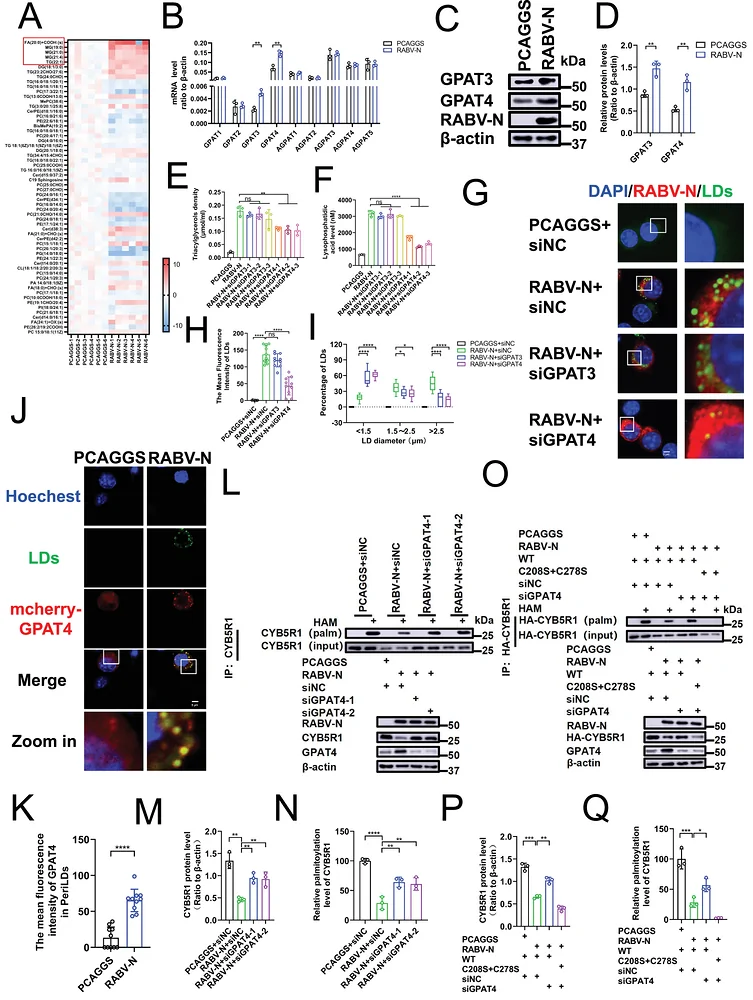
RABV‐N induces GPAT4 expression and translocation to LDs to promote LD maturation. (A) Lipidomics heatmap showing glyceride biosynthesis intermediates (FA/MG/DG/TG) in RABV‐N‐transfected N2a cells. (B) mRNA expression of GPAT and AGPAT family members in RABV‐N‐transfected N2a cells. Immunoblot analysis (C) and quantification (D) of GPAT3 and GPAT4 protein levels in RABV‐N‐transfected N2a cells. (E) TG and (F) lysophosphatidic acid (LPA) levels in RABV‐N‐transfected N2a cells with GPAT3/GPAT4 knockdown (siGPAT3/siGPAT4). LD staining in RABV‐N‐transfected N2a cells with GPAT3/GPAT4 knockdown (G, scale bar: 5 µm), quantification of LD number (H, *n* = 10 cells for each group), and LD diameter (I). (J) GPAT4‐mCherry (red) and BODIPY‐stained LDs (green) imaging in N2a cells co‐transfected with RABV‐N and mCherry‐GPAT4 (scale bar: 5 µm), (K) quantification of GPAT4 intensity in LD surface (n = 10 cells for each group). Immunoblot analysis (L) and quantification of CYB5R1 expression (M) and palmitoylation (N) in RABV‐N transfected N2a cells with siGPAT4. Immunoblot analysis (O) and quantification of mutated CYB5R1 expression (P) and palmitoylation (Q) in RABV‐N transfected N2a cells with siGPAT4. Representative results from three independent experiments are shown. Data are expressed as mean±SD, *n* = 3. Statistical significance was determined using unpaired two‐tailed student's t‐test or one‐way ANOVA followed by Tukey's multiple comparisons test. *p < 0.05, **p < 0.01, ***p < 0.001, ****p < 0.0001, ns: not significant.

Since RABV‐N reduces CYB5R1 stability by promoting LD formation, we further investigated the mechanism underlying RABV‐N‐induced LD biogenesis. Lipidomics sequencing of RABV‐N‐transfected N2a cells revealed significant accumulation of glyceride biosynthesis intermediates, including fatty acids, monoacylglycerol, diacylglycerol, and TG (Figure 6A), indicating that RABV‐N primarily drives glyceride synthesis to facilitate LD formation. Given that free fatty acids can be incorporated into TG synthesis via the Glycerol‐3‐phosphate Acyltransferases (GPAT) family, the rate‐limiting enzymes in TG biosynthesis [33], and that monoacylglycerol is generated during TG catabolism [34], we hypothesize that RABV‐N promotes LD biogenesis by enhancing the overall metabolic flux through the TG pathway. To test this, we examined the expression of nine rate‐limiting enzymes involved in TG synthesis, including GPATs and 1‐Acylglycerol‐3‐phosphate Acyltransferases. Only GPAT3 and GPAT4 showed significant upregulation at both mRNA and protein levels (Figure 6B–D).

GPAT3 and GPAT4 are key enzymes in lysophosphatidic acid (LPA) synthesis [35]. To dissect their roles in RABV‐N‐induced LD formation, we knocked down GPAT3 or GPAT4 using siRNA. GPAT4 knockdown alone significantly reduced RABV‐N‐induced TG and LPA accumulation (Figure 6E,F). Strikingly, GPAT3 knockdown decreased LD size without affecting LD number, whereas GPAT4 knockdown reduced both LD number and size (Figure 6G–I). Since GPAT4 has been shown to promote LD maturation by translocating to the LD surface during late‐stage LD formation [36], we assessed GPAT4 localization and found that RABV‐N also enhances GPAT4 translocation to the LD surface (Figure 6J,K). Additionally, we constructed a GPAT4 mutant (mCherry‐GPAT4 synthetic) that cannot target the surface of lipid droplets (LDs) based on the previous study [37]. Consistently, enhanced translocation of GPAT4 to the LD surface was also observed during RABV infection. Mutation of GPAT4 significantly reduced RABV‐induced LD biogenesis and the GPAT4 accumulation around LDs (Figure S6A–C). This impairment in LD formation was accompanied by markedly increased intracellular iron and lipid peroxidation levels (Figure S6D,E), which ultimately suppressed virus production (Figure S6F). These results indicate that RABV infection promotes the translocation of GPAT4 to the LD surface, which effectively facilitates LD formation and subsequently reduces cellular susceptibility to ferroptosis. Furthermore, GPAT4 knockdown rescued RABV‐N‐induced CYB5R1 depalmitoylation and restored CYB5R1 protein stability (Figure 6L–N), an effect abolished in CYB5R1 C208S+C278S mutants (Figure 6O–Q). Together, these findings demonstrate that RABV‐N induces LD formation and maturation by upregulating GPAT4 expression and promoting its translocation to the LD surface.

### 2.7 *Lyssavirus* genus N Proteins Conservedly Promote GPAT4‐Mediated LD Formation

**FIGURE 7:**
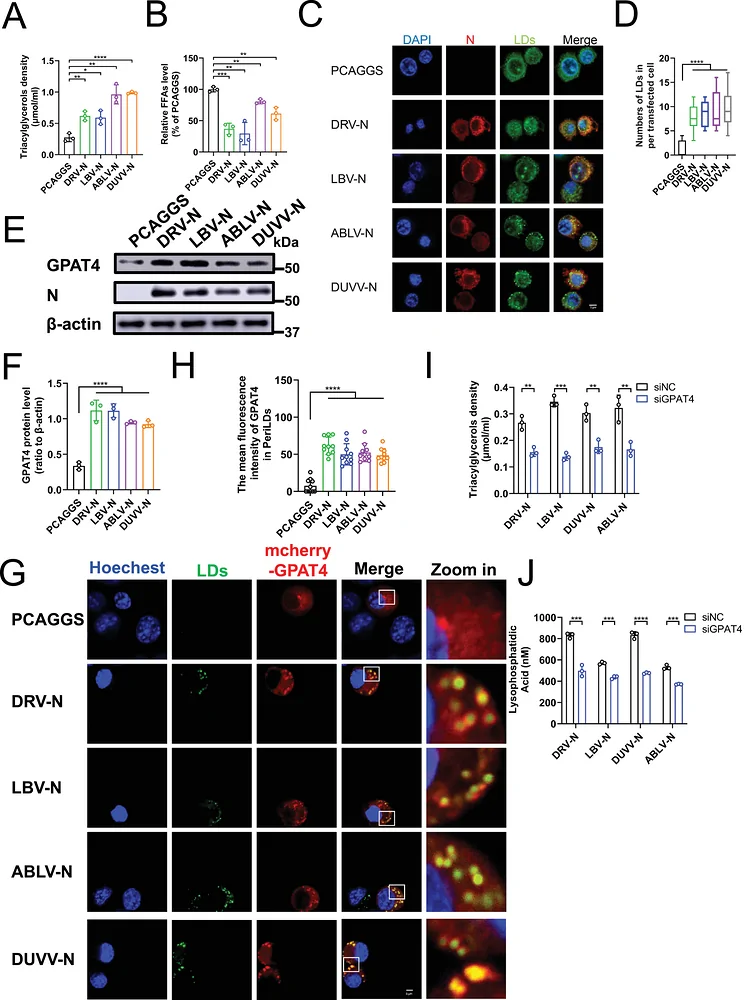
*Lyssavirus* genus N proteins conservedly promote GPAT4‐mediated LD formation. Quantification of TG (A) and FFA (B) levels in lyssavirus N‐transfected N2a cells. Representative image (C, scale bar: 5 µm) and quantification (D, *n* = 14 cells for each group) of LDs in N2a cells transfected with N proteins from diverse lyssaviruses (DRV, LBV, ABLV, DUVV). (E) Immunoblot analysis and (F) quantification of GPAT4 protein levels in lyssavirus N transfected N2a cells. Representative image (G, scale bar: 5 µm) and quantification the mean fluorescence intensity (H) of GPAT4 nearby LD surface in N2a cells cotransfected with PCAGGS (or DRV‐N, LBV‐N, ABLV‐N, DUVV‐N) and mCherry‐GPAT4 (n = 10 cells for each group). Quantification of TG (I) and LPA (J) levels in lyssavirus N transfected N2a cells ± siGPAT4. Representative results from three independent experiments are shown. Data are expressed as mean±SD, *n* = 3. Statistical significance was determined using unpaired two‐tailed student's *t*‐test or one‐way ANOVA followed by Tukey's multiple comparisons test. *p < 0.05, **p < 0.01, ***p < 0.001, ****p < 0.0001.

To determine whether the N protein‐mediated LD formation is conserved across the *Lyssavirus* genus, we transfected N2a cells with N proteins from diverse *lyssaviruses*: street rabies virus (DRV), LBV, ABLV, and DUVV. Transfection with each *lyssavirus* N protein significantly increased cellular TG levels and LD number, with a concomitant decrease in free fatty acid levels (Figure 7A–D). Consistently, all four N proteins upregulated GPAT4 expression and promoted its translocation to the LD surface (Figure 7E–H). Furthermore, siRNA‐mediated GPAT4 knockdown abrogated the *lyssavirus* N protein‐induced increases in TG and LPA levels (Figure 7I,J). Taken together, these finding demonstrate that GPAT4 upregulation is the conserved mechanism driving LD formation by *lyssavirus* N proteins.

### 2.8 *Lyssavirus* genus N Proteins Increase GPAT4‐Mediated LD Formation to Reduce CYB5R1‐Mediated Ferroptosis Susceptibility by Promoting APT1‐Mediated Depalmitoylation

**FIGURE 8:**
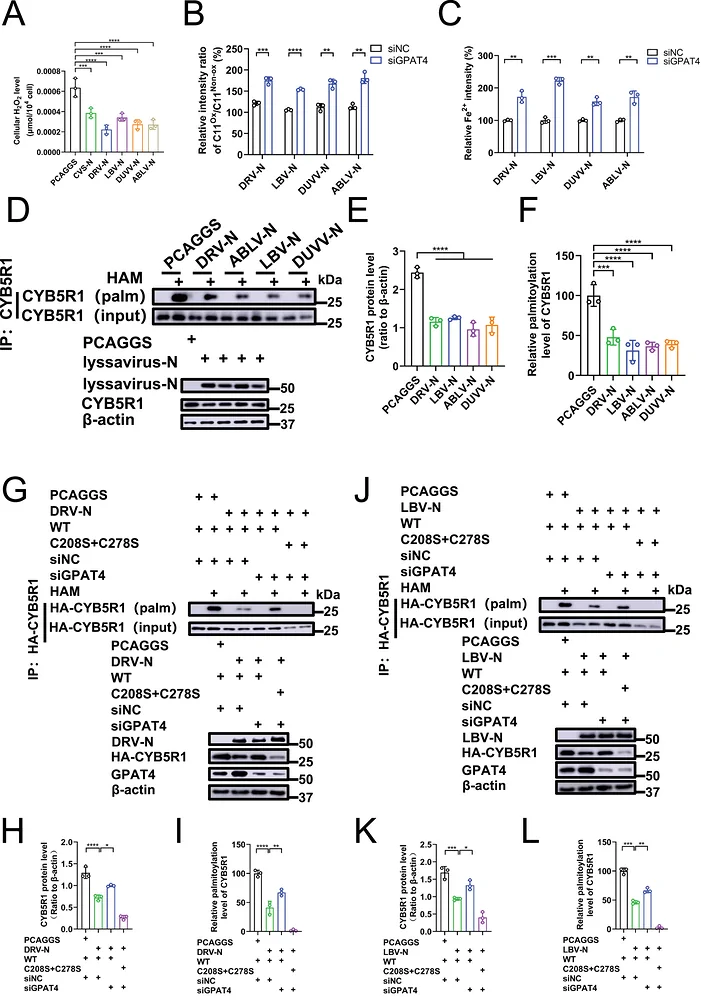
*Lyssavirus* genus N proteins increase GPAT4‐mediated LD formation to reduce CYB5R1‐mediated ferroptosis susceptibility by promoting APT1‐mediated depalmitoylation. (A) Quantification of H_2_O_2_ production in N2a cells transfected with N proteins from diverse *lyssaviruses* (DRV, LBV, ABLV, DUVV). Quantification of lipid peroxidation (B), and Fe^2+^ levels (C) in N2a cells transfected with siGPAT4. (D) Immunoblot analysis, quantification of CYB5R1 (E) expression and (F) palmitoylation in *lyssavirus* N transfected N2a cells. (G) Immunoblot analysis, quantification of CYB5R1 (H) expression and (I) palmitoylation in DRV‐N transfected N2a cells with indicated transfection. (J) Immunoblot analysis, quantification of CYB5R1 (K) expression and (L) palmitoylation in LBV‐N transfected N2a cells with indicated transfection. Representative results from three independent experiments are shown. Data are expressed as mean±SD, *n* = 3. Statistical significance was determined using unpaired two‐tailed student's t‐test or one‐way ANOVA followed by Tukey's multiple comparisons test. *, *P* < 0.05; **, *P* < 0.01; ***, *P* < 0.001, ****, *P* < 0.0001.

**FIGURE 9:**
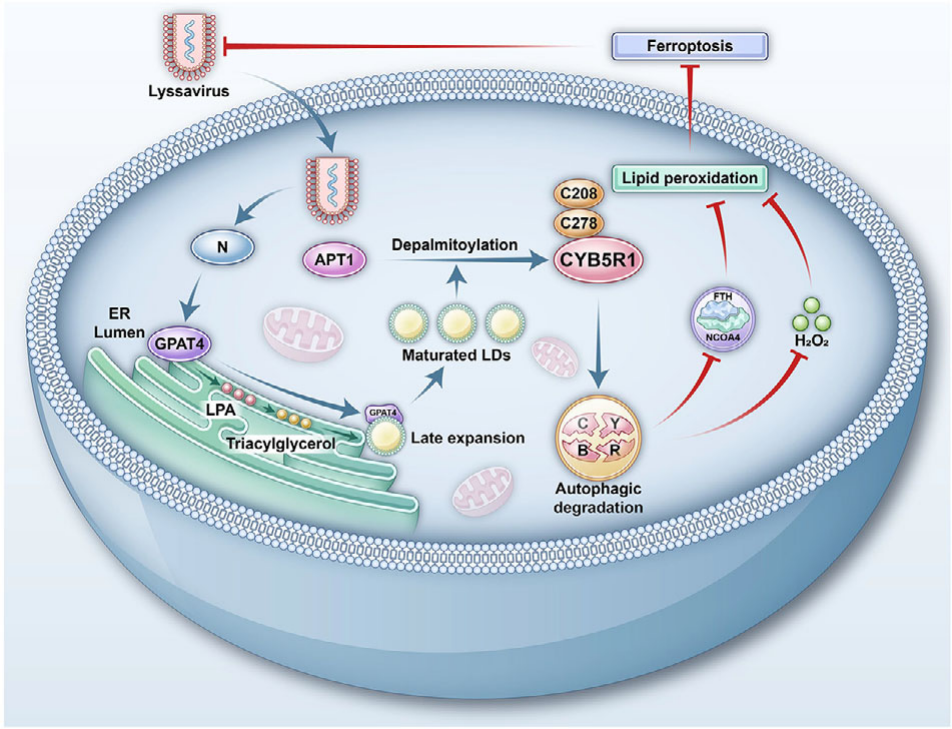
Schematic model of *lyssavirus* N protein‐mediated ferroptosis resistance via the GPAT4‐CYB5R1 Axis. *Lyssavirus* nucleoprotein (N) upregulates GPAT4 expression, thereby augmenting triacylglycerol (TG) biosynthesis and LD biogenesis. This metabolic reprogramming precipitates APT1‐mediated depalmitoylation of CYB5R1 at residues Cys208 and Cys278, facilitating its degradation via the autophagy‐lysosomal pathway. Concomitantly, this axis suppresses NCOA4‐mediated ferritinophagy, thereby attenuating ferroptosis susceptibility and ultimately fostering a permissive environment for viral replication.

Next, we evaluated whether *lyssavirus* N protein‐induced LD formation modulates ferroptosis. *Lyssavirus* N protein transfection significantly reduced H_2_O_2_ production (Figure 8A). Also, inhibiting LD formation with GPAT4 silencing in N protein‐transfected cells increased in lipid peroxidation and Fe^2+^ levels (Figure 8B,C), indicating that LD formation universally decreases ferroptosis susceptibility across *lyssaviruses*. Furthermore, all tested *lyssavirus* N proteins downregulated endogenous CYB5R1 expression and reduced its palmitoylation (Figure 8D–F). This effect was reversed by GPAT4 knockdown except in cells expressing CYB5R1 C208S+C278S mutants, where DRV and LBV N proteins failed to alter CYB5R1 stability (Figure 8G–L). Together, these findings demonstrate that N proteins across the *lyssavirus* genus share a conserved mechanism: they upregulate GPAT4 expression and promote its translocation to the LD surface, thereby reducing CYB5R1 stability through depalmitoylation at Cys208 and Cys278 (Figure 9).

## 3 Discussion

Viruses commonly induce LD biogenesis by modulating the expression of key lipid metabolic enzymes, thereby altering cellular lipid homeostasis. For instance, SARS‐CoV‐2 and Zika Virus infection upregulate Peroxisome Proliferator‐Activated Receptor gamma and Sterol Regulatory Element‐Binding Protein 1 to promote LD synthesis [38, 39]. Similarly, Bovine Viral Diarrhea Virus and Human Cytomegalovirus enhance LD formation by increasing the expression of Fatty Acid Synthase, Acetyl‐CoA Carboxylase 1, and Stearoyl‐CoA desaturase 1 [40, 41]. However, it remains unclear whether viral infection regulates the GPAT and AGPAT gene families, key enzymes in the LPA to phosphatidic acid to TG pathway, to influence LD biogenesis. Previous studies have shown that hepatitis C virus and SARS‐CoV‐2 manipulate the enzymatic activity of AGPAT1 and 2 to promote phosphatidic acid accumulation in double‐membrane vesicles, facilitating viral replication [42, 43]. Whether the GPAT‐mediated conversion of LPA to phosphatidic acid is also regulated during viral infection, thereby impacting TG synthesis, has not been established. In this study, we discovered that lyssavirus N protein promotes LPA synthesis and subsequent TG production by enhancing the expression and translocation of GPAT4, ultimately driving LD biogenesis. Furthermore, the generated LDs effectively reduce the palmitoylation of CYB5R1, thereby compromising its stability and ultimately inhibiting ferroptosis susceptibility. In summary, the lyssavirus N protein effectively promotes viral replication through the GPAT4‐LDs‐CYB5R1‐ferroptosis axis.

Recent studies have revealed the dual role of ferroptosis in viral infections. On one hand, the majority of viruses promote their own replication by inducing ferroptosis. For instance, the hemagglutinin protein of influenza A virus induces ferritinophagy to suppress interferon responses, thereby facilitating viral replication [44]. Similarly, pseudorabies virus disrupts iron homeostasis by activating transferrin receptor 1 and ferritinophagy, inducing ferroptosis to promote its replication [45]. Moreover, taurochenodeoxycholic acid inhibits SFTSV‐induced ferroptosis by upregulating fatty acid desaturase 2, thus suppressing viral replication [46]. On the other hand, ferroptosis can also serve as a host defense mechanism that significantly restricts viral replication. The ferroptosis agonist RSL3 has been shown to markedly inhibit the replication phase of porcine epidemic diarrhea virus [11]. Furthermore, zinc ions limit iridovirus replication by downregulating the expression and enzymatic activity of glutathione peroxidase 4, thereby triggering ferroptosis [12]. Additionally, fatty acid desaturase 2 inhibits hepatitis C virus replication by promoting ferroptosis through the conversion of saturated fatty acids into unsaturated ones [13].

Ferritinophagy, a selective autophagic process mediated by NCOA4, promotes ferritin degradation to release labile iron, which triggers Fenton reaction‐driven ROS production and subsequent ferroptosis [47, 48, 49, 50]. Our previous studies demonstrated that *lyssavirus* infection induces LD formation to facilitate viral budding and restricts arachidonic acid‐mediated ferroptosis, thereby promoting viral replication [28, 51]. Here, we expand this paradigm by identifying CYB5R1 as a critical mediator of ferroptosis susceptibility in *lyssavirus*‐infected neurons. Notably, CYB5R1 has been reported to donate electrons to multiple acceptor proteins, suggesting that CYB5R1 may intersect with other ferroptosis pathways beyond NCOA4 regulation [26]. Additionally, HECT domain‐containing E3 ubiquitin ligase HERC2 competitively binds FTH to inhibit NCOA4‐FTH complex formation [52, 53, 54, 55]. It raises the question: Does RABV‐induced LD biogenesis also modulate HERC2‐NCOA4 interplay to fine‐tune ferritinophagy? Therefore, it is worth further investigating the relationship between LDs and HERC2 to address this gap, potentially revealing a “lipid‐iron” regulatory hub in viral infection. PTMs are pivotal for protein functional plasticity, with palmitoylation emerging as a key modulator of protein stability and subcellular localization [56, 57, 58]. For instance, site‐specific palmitoylation of GPX4 [59], B7‐H4 [60], and TIM‐3 [61] enhances their stability to promote tumor immune evasion. Additionally, palmitoylation regulates signaling pathways, such as transferrin receptor 1 (TfR1) and dual leucine zipper kinase (DLK), whose palmitoylation affects the cell fate of neurons in ischemic stroke and neurodegenerative processes [62, 63]. Our study uncovers a novel viral strategy: *lyssavirus* N protein targets CYB5R1 palmitoylation at Cys208 and Cys278 to trigger its degradation. The inability of CYB5R1 C208S+C278S mutants to restore ferroptosis confirms these residues as “molecular switches” governing antiviral defense, a finding with therapeutic implications. Pharmacological stabilization of CYB5R1 palmitoylation (e.g., via APT1 inhibitors like ML348) could potentially restrict viral replication in the brain, though this requires validation in animal models of rabies encephalitis, which is warranted in our future study.

As two acyl‐protein thioesterases, APT1 and APT2 are localized to the mitochondria and Golgi apparatus, respectively, and are widely distributed across tissues [30]. They are responsible for depalmitoylation to affect protein stability, but the regulatory mechanisms remain incompletely understood. APT1 mediated depalmitoylation could promote renal fibrosis and affect the recovery cycle from inflammatory pain in mice [25, 64]. In colorectal cancer models, knockout of APT2 significantly accelerated cancer progression [65]. Our observation that only APT1 modulates *lyssavirus* N protein‐induced CYB5R1 depalmitoylation suggests substrate specificity, which is possibly due to higher binding affinity between APT1 and CYB5R1's C‐terminal domain. It is noteworthy that the palmitoylation sites and structural features of substrate proteins influence the binding and catalytic efficiency of APTs [66, 67]. Whether *lyssavirus* N protein regulates APT1 expression levels or its PTMs also requires deeper exploration in subsequent studies.

Glycerol‐3‐phosphate acyltransferases 3 and 4 (GPAT3/4) are rate‐limiting enzymes in glycerolipid synthesis, catalyzing lysophosphatidic acid production and translocating to LD surfaces to promote maturation [35, 68, 69, 70]. Recent studies show CHP1 stabilizes GPAT3/4 and counteracts Seipin‐mediated LD size restriction [35]. Prior studies have shown that viruses exploit LDs for replication and immune evasion [71, 72], but the molecular link between LD biogenesis in viral infection and ferroptosis regulation remained elusive. Our identification of GPAT4 as a dual regulator of LD formation and protein palmitoylation fills this gap. By sequestering free fatty acids, *lyssavirus* N‐induced LDs limit substrate availability for CYB5R1 palmitoylation—a novel mechanism distinct from canonical viral strategies targeting ferroptosis enzymes [73, 74, 75]. However, knockdown of GPAT3 only restricted LD size without significantly affecting TG synthesis or overall LD formation. The differential effects of GPAT3/4 knockdown, i.e., GPAT3 depletion only reduces LD size, while GPAT4 loss abrogates LD formation, likely reflect higher basal GPAT4 expression in neuronal cells.

In summary, we establish a conserved regulatory network wherein *lyssavirus* N protein hijacks GPAT4‐dependent LD biogenesis to suppress CYB5R1‐mediated ferroptosis. This pathway, conserved across *lyssavirus* genus, suggests LD‐mediated palmitoylation suppression is a core viral adaptation. Future studies should explore whether this mechanism extends to other enveloped viruses (e.g., flaviviruses, coronaviruses) and evaluate APT1/GPAT4 inhibitors as broad‐spectrum antiviral agents.

## 4 Methods

### 4.1 Animals, Cells, Antibodies, Chemical Inhibitors, and Reagents

The N2a (ATCC CCL‐131) and SK‐N‐SH (ATCC HTB‐11) cell lines were sourced from the American Type Culture Collection (ATCC). Primary neuronal cultures were isolated and maintained as previously described [10]. Dulbecco's Modified Eagle Medium and fetal bovine serum were procured from Gibco. Antibodies utilized in this study include: anti‐β‐actin (AC026), anti‐FTH1 (A25458PM), anti‐HA (AE105), anti‐LC3B (A19665), anti‐Flag (AE005), anti‐V5 (AE017), anti‐myc (AE010), anti‐APT1 (A26848) and ABflo 594 anti‐LAMP1 (A24364) from ABclonal (Wuhan, China); anti‐CYB5R1 (11807‐1‐AP), anti‐GPAT3 (20603‐1‐AP) and anti‐GPAT4 (16762‐1‐AP) from Proteintech (Wuhan, China); anti‐NCOA4 (ab314554) from abcam; HRP‐conjugated goat anti‐mouse antibody (BA1051) and anti‐rabbit antibody (BA1055) from Boster (Wuhan, China); DyLight 594 goat anti‐mouse IgG (H+L) cross‐adsorbed secondary antibody (35511) and Alexa Fluor 488 goat anti‐mouse IgG (H+L) cross‐adsorbed secondary antibody (11001) from Invitrogen; BODIPY 493/503 (GC42959) from GLPBIO (CA, USA); 2‐Bromohexadecanoic acid (T35364), MG132 (T2154), Biotin‐HPDP (T19955), Hydroxyamine hydrochloride (T11587), Chloroquine (T8689), ML‐348 (T3439) and ML‐349 (T16108) from TargetMol (MA, USA); Protein G Magnetic Beads (HY‐K0204), A922500 (HY‐10038) and PF‐06424439 (HY‐108341A) from MedChemExpress (NJ, USA); MDA Assay Kit (M496), BDP 581/591 C11 (L267) and FerroOrange (F374) from DoJindo; Hydrogen Peroxide Assay Kit (S0038), Penicillin‐streptomycin (C0222), PI (ST511), Western/IP lysis buffer (C0013), Triacylglycerol ELISA Kit (S0219S) and Hoechst33342 (C1026) from Beyotime (Shanghai, China); Lysophosphatidic Acid ELISA Kit (YJ923690) from YUANJU Bio (Shanghai, China); HiScript II first Strand cDNA Synthesis Kit (R211‐01), HiScript III first Strand cDNA Synthesis Kit (R222‐01) and ChamQ SYBR qPCR Master Mix (Q711‐02) from Vazyme (Nanjing, China); Prolong Gold antifade mounting solution (P10144) from Invitrogen.

### 4.2 Cell Culture, Transfection, and Infection

N2a (ATCC CCL‐131) and SK‐N‐SH (ATCC HTB‐11) cells were maintained in Dulbecco's Modified Eagle Medium supplemented with 10% fetal bovine serum and 1% penicillin‐streptomycin at 37°C in a humidified atmosphere containing 5% CO_2_. After transfection and infection, cells maintained with DMEM containing 2% FBS and 1% penicillin‐streptomycin for the duration of the experiment.

### 4.3 CYB5R1 Knockout N2a Cell Line Construction

Three target sgRNAs was designed and cloned into px458‐Cas9‐puro vector (CYB5R1‐sgRNA‐1: GGGTCCTGGAGAGTAACCTGCGG, CYB5R1‐sgRNA‐2: CAGTGA

GCCACAACACCAGGAGG, CYB5R1‐sgRNA‐3: GTCACCAGTGATGAAGACCAAGG). The recombinant plasmids were then transfected into N2a cells, and the positive cells were then separated with a flow cytometer and cultured which was used for the experiments.

### 4.4 Plasmid Construction and Sh/siRNA Transfection

Murine CYB5R1 (WT, C208S, C278S, C288S), NCOA4, FTH, RABV structural proteins (N, P, M, G), *lyssavirus* N protein (DRV, LBV, ABLV and DUVV) were cloned into the pCAGGS vector (the chicken β‐actin/CMV enhancer (CAG) promoter) expressing HA, Flag or myc tag, respectively. Three short harpin of RNAs (shRNA), three small types of small interfering RNAs (siRNA) targeting GPAT4 and APT1 were designed and provided in Table S1. Transfection of shRNA, siRNA or plasmids were performed using the Lipofectamine 3000 reagent (Thermo Fisher Scientific) according to the manufacturer’ s instructions.

### 4.5 Triacylglycerol and Lysophosphatidic Acid ELISA

Collected cells were harvested, washed with ice‐cold phosphate‐buffered saline (PBS), and centrifuged. Cellular TG and lysophosphatidic acid (LPA) content were subsequently quantified using commercial ELISA kits according to the manufacturer's protocol. Absorbance (optical density, OD) was measured at 450 nm using a SpectraMax 190 microplate reader (Molecular Devices, CA). TG and LPA concentrations were calculated from concurrently generated standard curves.

### 4.6 RNA‐seq Analysis

Total RNA was isolated using TRIzol Reagent following the manufacturer's protocol. Stranded RNA sequencing libraries were subsequently prepared on the BGI sequencing platform according to the manufacturer's specifications. The resulting RNA‐seq reads were aligned to the GRCm39.112 reference genome assembly. Differential gene expression analysis was conducted using DESeq2 (v1.26.0), and the resulting *p* values were adjusted for multiple testing using the Benjamini‐Hochberg method to control the false discovery rate (FDR).

### 4.7 Quantitative PCR Analysis

Purified RNA served as the template to reverse cDNA, and subsequently analyzed by quantitative PCR (qPCR) on a QuantStudio 7 Real‐Time PCR System (Applied Biosystems, USA) under the following thermocycling conditions: initial denaturation at 95°C for 2 min; followed by 40 cycles of denaturation at 95°C for 5 s and annealing/extension at 60°C for 30 s. Relative gene expression levels were calculated and normalized to an endogenous control gene. Primer sequences are detailed in Table S1.

### 4.8 Lipidomic Analysis

Collected cells were harvested and methanol (0.75 mL) was added to samples, which were placed into a glass tube with a Teflon lined cap, and the tube was vortexed. Subsequently, 2.5 mL of MTBE was added and the mixture was incubated for 1 h at room temperature in a shaker. Phase separation was induced by adding 0.625 mL of MS‐grade water. Upon 10 min of incubation at room temperature, the sample was centrifuged at 1000 g for 10 min. The upper (organic) phase was collected, and the lower phase was reextracted with 1 mL of the solvent mixture (MTBE/methanol/water (10:3:2.5, v/v/v)), and collecting the upper phase. Combined organic phases were dried and dissolved in 100 µl of isopropanol for storage. Then analyzed by LC‐MS/MS. UHPLC‐MS/MS analyses were performed using a Vanquish UHPLC system (Thermo Fisher Scientific) coupled with an Orbitrap Q ExactiveTM HF mass spectrometer (Thermo Fisher Scientific).

### 4.9 Viral Attachment, Entry, and Replication Analysis

To assess viral attachment, N2a cells transfected with CYB5R1 or an empty vector were infected with RABV (MOI = 1) at 4°C for 1 h to allow attachment without internalization. The cells were then washed three times with ice‐cold PBS to remove unbound virus, and total RNA was immediately extracted for subsequent analysis. To assess viral entry, N2a cells transfected with CYB5R1 or an empty vector were infected with RABV (MOI = 1) at 37°C for 2 h. Following infection, the cells were washed three times with PBS and total RNA was immediately extracted. For the replication assay, N2a cells transfected with CYB5R1 or an empty vector were infected with RABV (MOI = 1) at 37°C for 24 h, washed three times with PBS, and total RNA was immediately extracted. Viral genomic RNA copies and RABV‐N gene were quantified by RT‐qPCR, the specific primer sequences are listed in Table S1.

### 4.10 Western Blotting

Following washing with ice‐cold phosphate‐buffered saline (PBS), cells were lysed in RIPA buffer and centrifuged at 12,000 g at 4°C. Supernatants were combined with SDS‐PAGE loading buffer, denatured by boiling, resolved by SDS‐PAGE and transferred onto polyvinylidene difluoride (PVDF) membranes. Membranes were blocked for 1 h with 5% (w/v) non‐fat dry milk, incubating with primary antibodies overnight at 4°C. After three washes with Tris‐buffered saline containing 0.1% Tween‐20 (TBST), membranes were incubated with horseradish peroxidase (HRP)‐conjugated secondary antibodies for 1 h at room temperature. Immunoreactive bands were finally detected using enhanced chemiluminescence (ECL) substrate and visualized with an Amersham Imager 600 (GE Healthcare Life Sciences).

### 4.11 Cell Death Assay via Propidium Iodide Staining

Infected cells were harvested, washed with ice‐cold phosphate‐buffered saline (PBS), and stained with propidium iodide (PI) for 30 min. After two washes with PBS, cell counts were determined using a Countstar automated cell counter. Subsequently, 10^4^ cells per sample were processed to quantify PI fluorescence intensity using a fluorescent microplate reader (Tecan).

### 4.12 Virus Titration

Viral titers of RABV were determined by using a direct fluorescence assay as previously described [76]. Supernatants were diluted 10‐fold into plates in quadruplicate, and BSR cells were added and incubated at 37°C for 48 h. Plates were fixed with 80% cold acetone for 2 h at ‐20°C, and incubated with FITC‐RABV‐P antibody for 1 h at 37°C. The positive foci were counted by a fluorescence microscope (ZEISS, Germany), virus titers were calculated and expressed as FFU/mL.

### 4.13 Immunofluorescence Analysis

N2a cells were transfected with CYB5R1 and infected with RABV. Cells were fixed with 80% cold acetone for 2 h at −20°C, and incubated with FITC‐RABV‐P antibody for 1 h at 37°C. DAPI was used to stain nuclei at room temperature for 10 min. Cells were incubated with Prolong Gold antifade mounting solution to prevent quenching and imaged with a confocal microscope (ZEISS, Germany).

### 4.14 Confocal Microscopy

N2a cells were cultured in polylysine‐pretreated coverslips, transfected with CYB5R1, GFP‐mCherry LC3, or *lyssavirus* N protein plasmids at 48 h. Following fixation with 4% paraformaldehyde at room temperature (RT) for 30 min, cells were quenched with PBS containing 0.1 M glycine for 15 min and washed with PBS. Subsequent permeabilization was performed using 0.1% Triton X‐100 in PBS for 30 min. Blocking with blocking buffer (10 mM Tris‐HCl, pH 7.5, 150 mM NaCl, 2% bovine serum albumin (BSA), 10% goat serum) for 30 min and washing with PBS, cells were incubated with primary antibody for 2 h including LAMP1, FTH1, GPAT4 or HA‐tag. Secondary antibodies conjugated to Alexa Fluor 488, 594, or 647 were used to incubated cells for 2 h at room temperature and Nuclei were stained with DAPI for 5 min. Coverslips were mounted with ProLong Gold Antifade Mountant to minimize photobleaching and imaged on a Nikon confocal laser scanning microscope. Fluorescence excitation was achieved using 405 nm (DAPI), 488 nm (Alexa Fluor 488), 561 nm (Alexa Fluor 594), and 633 nm (Alexa Fluor 647) laser lines.

To investigate GPAT4 translocation, N2a cells were transfected with mCherry‐GPAT4 and multiple lyssavirus N plasmids (RABV, DRV, LBV, ABLV, DUVV) for 48 h. Subsequently, cells were washed with PBS, stained with BODIPY 493/503 for 30 min, washed again with PBS, and then stained with Hoechst 33342 for 5 min. Following a final PBS wash, cells were imaged using a Nikon confocal laser scanning microscope.

### 4.15 Lipid Peroxidation, Fe^2+^, ROS, and 4‐HNE Assay

Probes of BDP 581/591 C11 (non‐oxidized, Ex: 550 nm, Em: 590 nm; oxidized, Ex: 480 nm, Em: 530 nm), FerroOrange (Ex: 543 nm, Em: 580 nm), DCFH‐DA (Ex: 488 nm, Em: 525 nm), and 4‐HNE antibody were used to determined cellular lipid peroxidation, Fe^2+^, ROS and 4‐HNE, respectively. N2a or SK‐N‐SH cells were transfected with a plasmid expressing CYB5R1 and infected with RABV (MOI = 1, 48 h). No exogenous iron was added to the culture media. Cells were harvested, washed twice with ice‐cold phosphate‐buffered saline (PBS), and incubated with the respective detection probes diluted in serum‐free Hank's Balanced Salt Solution (e.g., BDP 581/591 C11, FerroOrange, or DCFH‐DA) for 30 min at 37°C. For 4‐HNE determination, cells were incubated with the primary anti‐4‐HNE antibody for 60 min and subsequently with the Alexa Fluor 488 secondary antibody for 45 min at 37°C. Following PBS washes, cell suspensions were counted using a Countstar automated cell counter. 10^4^ cells per sample were analyzed using a fluorescent microplate reader (Tecan).

### 4.16 Co‐Immunoprecipitation (Co‐IP)

Cultured cells were washed sequentially with ice‐cold phosphate‐buffered saline (PBS) and lysed in Western/IP lysis buffer supplemented with protease/phosphatase inhibitor cocktails for 45 min at 4°C under gentle agitation. Lysates were centrifuged at 12 000 g for 10 min at 4°C, and supernatants were collected. Protein A/G magnetic beads were conjugated with target‐specific primary antibodies or species‐matched IgG isotype controls at room temperature for 45 min with gentle agitation. Following three washes with lysis buffer, the antibody‐bound beads were incubated with supernatants overnight at 4°C. Immunocomplex‐bound beads were washed five times with lysis buffer, resuspended in SDS loading buffer, and denatured at 95°C for 8 min. Eluted proteins were subsequently resolved by SDS‐PAGE for immunoblot analysis.

### 4.17 MDA Assay

N2a cells were transfected with CYB5R1 infected with RABV (MOI = 1, 48 h). Cells were harvested and washed with ice‐cold phosphate‐buffered saline (PBS) and counted with a Countstar automated cell counter. Cells were resuspends and lysed to determine cellular MDA according to the manufacturer's instructions. The thiobarbituric acid was added to form a TBA‐MDA mixture and determined via a fluorescent microplate reader (TECAN) (Ex: 540 nm, Em: 590 nm).

### 4.18 Palmitoyl‐CoA Assay

Collected N2a cells were first washed with PBS, and LDs were subsequently isolated from the cells following the instructions of the Lipid Droplet Isolation Kit (Abcam, ab242290). After centrifugation, the upper LD fraction and the lower aqueous phase were collected separately. The palmitoyl‐CoA level in the lower aqueous phase was then measured by Enzyme‐linked Palmitoyl‐CoA Detection Kit (mlbio, ml959002).

### 4.19 Acyl‐biotin Exchange (ABE) Assay

The acyl‐biotin exchange (ABE) assay was conducted as previously described with modifications [77]. Cultured cells were washed twice with ice‐cold phosphate‐buffered saline (PBS) and lysed in Western/IP lysis buffer supplemented with protease/phosphatase inhibitor cocktails for 45 min at 4°C with gentle agitation. Lysates were centrifuged at 12,000 g for 10 min at 4°C. Supernatants were incubated in solubilization buffer (4% SDS, 1.7% Triton X‐100, 50 mM Tris pH 8.0, 5 mM EDTA, 20 mM methyl methanethiosulfonate (MMTS) containing protease/phosphatase inhibitors at 40°C for 3 h. Protein A/G magnetic beads were conjugated with anti‐CYB5R1 primary antibodies at room temperature for 45 min under agitation. Antibody‐bound beads were washed sequentially: five times with pH 7.5 lysis buffer and three times with pH 7.2 lysis buffer. Beads were then incubated in pH 7.2 lysis buffer containing 1 M hydroxylamine (HAM) with protease/phosphatase inhibitors for 1 h at room temperature. Parallel samples were processed with (+HAM) or without (‐HAM) hydroxylamine treatment. Following hydroxylamine exposure, beads were washed: four times with pH 7.2 lysis buffer and once with pH 6.2 lysis buffer. Washed beads were incubated with HPDP‐biotin in pH 6.2 lysis buffer at 4°C for 1 h. After final washes (once with pH 6.2 buffer, three times with pH 7.5 buffer), samples were resolved by SDS‐PAGE and analyzed by immunoblotting using target‐specific antibodies.

### 4.20 Statistical Analysis

Data were analyzed using GraphPad Prism 9 (GraphPad Software, San Diego, CA). The gray band ratio of immunoblots and mean fluorescence intensity were calculated using ImageJ software (https://imagej.nih.gov/ij/). Data are presented as mean±SD, and the sample size (n) for each experiment is indicated in the corresponding figure legends. Statistical significance was assessed using an unpaired two‐tailed t‐test, one‐way ANOVA followed by Tukey's multiple comparisons test, or two‐way ANOVA followed by Tukey's multiple comparisons test, as appropriate. Significance levels are indicated as follows: *P < 0.05, **P < 0.01, ***P < 0.001, ****P < 0.0001, ns, not significant.

## Author Contributions

J.Z.: conceptualization, investigation, funding acquisition, writing – original draft, methodology, formal analysis, resources, project administration. C.L.: visualization, validation, investigation, software, resources, methodology. P.C.: data curation, software, validation, visualization, resources, investigation. M.S.: investigation, methodology, software, resources, data curation, formal analysis, visualization. Z.F.F.: supervision, writing – review & editing, project administration, conceptualization. L.Z.: writing – review & editing, conceptualization, supervision, project administration. M.Z.: supervision, conceptualization, writing – review & editing, funding acquisition, project administration.

## Ethics Approvals

Animal experiments were conducted in strict accordance with approved protocols from the Scientific Ethics Committee of Huazhong Agricultural University (Permit Number: 202412260013). All institutional and national guidelines governing the care and use of laboratory animals were rigorously adhered.

## Conflicts of Interest

The authors declare no conflicts of interest.

## Supporting information




**Supporting File**: advs77672‐sup‐0001‐SuppMat.docx.

## Data Availability

The data that support the findings of this study are available from the corresponding author upon reasonable request.
