# Supplementary material for: Lyssavirus Nucleoprotein Induces GPAT4‐Mediated CYB5R1 Depalmitoylation to Suppress Ferroptosis and Promote Viral Replication
Source: Adv Sci (Weinh). 2026 Sep 9:e77672. Online ahead of print. doi: 10.1002/advs.77672 (PMC13558913; doi:10.1002/advs.77672)
Supplement: Supplementary file 1 [file ADVS-9999-e77672-s001.docx]

**Changes are highlighted in yellow.**

**Supplementary Information for**

**Lyssavirus Nucleoprotein Induces GPAT4-Mediated CYB5R1 Depalmitoylation to Suppress Ferroptosis and Promote Viral Replication**

Jianqing Zhao^1,3,4^, Chuan Liang^1,3,4^, Pingping Chen^1,3,4^, Meixin Sun^1,3,4^, Zhen F Fu^1,3,4^, Ling Zhao^1,2,3,4^*, Ming Zhou^1,3,4^*

^1^National Key Laboratory of Agricultural Microbiology, Huazhong Agricultural University, Wuhan 430070, China.

^2^Hubei Hongshan Laboratory, Wuhan 430070, China.

^3^Key Laboratory of Preventive Veterinary Medicine of Hubei Province, College of Veterinary Medicine, Huazhong Agricultural University, Wuhan 430070, China.

^4^Frontiers Science Center for Animal Breeding and Sustainable Production, Wuhan 430070, China.

*Corresponding Authors:

Ming Zhou, mikchail@163.com; mingzhou@mail.hzau.edu.cn.

Ling Zhao, zling604@outlook.com; lingzhao@mail.hzau.edu.cn.

**Fig. S1** Overexpression CYB5R1 enhances ferroptosis susceptibility in neuronal cell. Quantification of cell death (A), MDA (B), Fe^2+^ (C) and lipid peroxidation (D) in N2a cells transfected with PCAGGS or CYB5R1 and infected with or without RABV (MOI=1, 48 h). Quantification of cell death (E), MDA (F), Fe^2+^ (G) and lipid peroxidation (H) in SK-N-SH cells transfected with PCAGGS or CYB5R1 and infected with or without RABV (MOI=1, 48 h). Quantification of cell death in PCAGGS- or CYB5R1-transfected N2a cells (I) and SK-N-SH cells (J) following RABV infection with Ferrostatin-1 (10 μM), Z-VAD-FMK (25 μM) or GSK872 (2.5 μM) treatment. Representative results from three independent experiments are shown. Data are expressed as mean±SD, n=3. Statistical significance was determined using unpaired two-tailed student’s t-test. *, *P*<0.05; **, *P*<0.01; ***, *P*<0.001, ****, *P*<0.0001.

**Fig. S2** Loss of CYB5R1 abrogates the effect of LD restriction on viral replication. (A) Viral titers in CYB5R1 WT and KO N2a cells treated with or without DGAT inhibitors (A922500+PF06424439; 8 μg/mL+ 10 μg/mL) and infected with RABV (MOI=1, 48 h). Quantification of lipid peroxidation (B) and Fe^2+^ (C) in N2a cells treated with or without DGAT inhibitors and infected with RABV (MOI=1, 48 h). Representative results from three independent experiments are shown. Data are expressed as mean±SD, n=3. Statistical significance was determined using two-way ANOVA followed by Tukey's multiple comparisons test. *, *P*<0.05; **, *P*<0.01; ***, *P*<0.001, ****, *P*<0.0001.

**Fig. S3** DGAT inhibition increases NCOA4 expression and decreases FTH expression in RABV infection. (A) Quantification of lipid metabolism related genes mRNA expression in RABV infected (MOI=1, 48 h) N2a cells treated with DMSO or DGAT inhibitor (A922500+PF06424439, 8 μg/mL+ 10 μg/mL). Immunoblot analysis (B) and quantification (C) of NCOA4 and FTH expression in RABV infected N2a cells. Representative results from three independent experiments are shown. Data are expressed as mean±SD, n=3. Statistical significance was determined using unpaired two-tailed student's t test. **, *P*<0.01, ***, *P*<0.001.

**Fig. S4** RABV infection induces LD generation to decrease cytosolic palmitoyl-CoA content. Quantification of cellular palmitoyl-CoA in RABV-infected N2a cells (MOI=1, 48 h) treated with or without DGATi (A922500+PF06424439, 8 μg/mL+ 10 μg/mL). Representative results from three independent experiments are shown. Data are expressed as mean±SD, n=3. Statistical significance was determined using unpaired two-tailed student's t test. ***, *P*<0.01, ****, *P*<0.0001.

**Fig. S5** RABV-N modulates CYB5R1 palmitoylation by increasing APT1 mediated depalmitoylation. Immunoblot analysis (A) and quantification of exogenous CYB5R1 expression (B) in RABV-N transfected N2a cells. Quantification of mRNA expression (C), Immunoblot analysis (D) and quantification (E) of CYB5R1 expression in RABV-N transfected N2a cells treated with ML348 in gradient concentration. Immunoblot analysis (F) and quantification (G) of APT1 expression in RABV-N transfected N2a cells treated with DGATi (A922500+PF06424439, 8 μg/mL+ 10 μg/mL). Immunoblot analysis (H) and quantification (G) of APT1 expression in RABV-N transfected N2a cells treated with 2-BP in gradient concentration. Representative results from three independent experiments are shown. Data are expressed as mean±SD, n=3. Statistical significance was determined using unpaired two-tailed student's t test or one-way ANOVA followed by Tukey's multiple comparisons test. *, *P*<0.05; **, *P*<0.01; ***, *P*<0.01; ****, *P*<0.0001, ns, not significant.

**Fig. S6** RABV infection induces GPAT4 translocation to the surface of LDs. Representative image (A, scale bar: 5 μm) and quantification of LDs (B) and GPAT4 fluorescence nearby LDs (C) in RABV-infected N2a cells (MOI=1, 48 h) transfected with mCherry-GPAT4 (WT) or mCherry-GPAT4 (synthetic), n=10 cells for each group. Quantification of Fe^2+^ (D) and lipid peroxidation (E) in RABV-infected N2a cells transfected with GPAT4 (WT) or GPAT4 (synthetic). (F) Viral titers in N2a cells transfected with GPAT4 WT or GPAT4 synthetic and infected with RABV. Representative results from three independent experiments are shown. Data are expressed as mean±SD, n=3. Statistical significance was determined using unpaired two-tailed student's t test. **, *P*<0.01, ***, *P*<0.001, ****, *P*<0.0001.

**Table S1 Primers used in the study.**

A. **Primers for qPCR**

| Primer | Sequences (5' to 3') |
| --- | --- |
| β-actin-F | AGAGGGAAATCGTGCGTGAC |
| β-actin-R | CAATAGTGATGACCTGGCCGT |
| PLIN2-F | GGAGGAAAGACTGCCTATTCTGA |
| PLIN2-R | CATCCTTCGCCCCAGTTACG |
| CYB5R1-F | CTGATCCGGGCCATCTTGAAA |
| CYB5R1-R | CTAGGTCCTCCCGCAGGATTA |
| TFR-F | GTTTCTGCCAGCCCCTTATTAT |
| TFR-R | GCAAGGAAAGGATATGCAGCA |
| DMT-F | CAATGTCTTTGTCGTGTCCGT |
| DMT-R | GCGACCATTTTAGGTTCAGGAAT |
| IRP1-F | AGAACCCATTTGCACACCTTG |
| IRP1-R | AGCGTCCGTATCTTGAGTCCT |
| IRP2-F | TTCTGCCTTACTCAATACGGGT |
| IRP2-R | AGGGCACTTCAACATTGCTCT |
| FPN-F | ACCAAGGCAAGAGATCAAACC |
| FPN-R | AGACACTGCAAAGTGCCACAT |
| FTH-F | CAAGTGCGCCAGAACTACCA |
| FTH-R | GCCACATCATCTCGGTCAAAA |
| FTL-F | CCATCTGACCAACCTCCGC |
| FTL-R | CGCTCAAAGAGATACTCGCC |
| NCOA4-F | GCCCTACAATGTGAGTGATTGG |
| NCOA4-R | ACTGGTGCAAGGCTCGTTG |
| GPAT1-F | ACAGTTGGCACAATAGACGTTT |
| GPAT1-R | CCTTCCATTTCAGTGTTGCAGA |
| GPAT2-F | CACTGCTCCGAGGTTTTGATG |
| GPAT2-R | AGGTTGGCAGCAATTCCATAC |
| GPAT3-F | GGCCTTCGGATTATCCCTGG |
| GPAT3-R | CTTGGGGGCTCCTTTCTGAA |
| GPAT4-F | AGCTTGATTGTCAACCTCCTG |
| GPAT4-R | CCGTTGGTGTAGGGCTTGT |
| AGPAT1-F | TAAGATGGCCTTCTACAACGGC |
| AGPAT1-R | CCATACAGGTATTTGACGTGGAG |
| AGPAT2-F | CAGCCAGGTTCTACGCCAAG |
| AGPAT2-R | TGATGCTCATGTTATCCACGGT |
| AGPAT3-F | CTGCTTGCCTACCTGAAGACC |
| AGPAT3-R | GATACGGCGGTATAGGTGCTT |
| AGPAT4-F | CCAGTTTCTATGTCACCTGGTC |
| AGPAT4-R | GCAGAGTCTGGCATTGATCTTG |
| AGPAT5-F | CACACGTACTCTATGCGCTAC |
| AGPAT5-R | AAGAAGAGCACCATGTTCTGG |

B. **Primers for qPCR (viral RNA)**

| Primer | Sequences (5' to 3') |
| --- | --- |
| N-mRNA-F | GATCGTGGAACACCATACCC |
| N-mRNA-R | TTCATAAGCGGTGACGACTG |
| vRNA-F | ACAGGCAACACCACTGATAA |
| vRNA-R | TCAGCGGGACATATTCAGGA |

C. **Sequence for shRNA/siRNA**

| Primer | Sequences (5' to 3') |
| --- | --- |
| shNCOA4-1 | GGAAAGGACAAGAATGGAATG |
| shNCOA4-2 | TTCGGGCTGAACAGCAAATTA |
| shNCOA4-3 | CACAGGAAGCATTTCTTAATT |
| siGPAT4-1 | UCUGUAUUGUCAGAAGUACCA |
| siGPAT4-2 | UAAAGAUCUGUAUUGUCAGAA |
| siGPAT4-3 | AUUAAGUGAACGUGUUUACUC |
| siAPT1-1 | CUUACUGUUGAAAGACUAA |
| siAPT1-2 | GAUCAACAGUGCUAAUCGA |
| siAPT1-3 | GGAUGUCAAGCACUUCAUU |
